# Variations on gut health and energy metabolism in pigs and humans by intake of different dietary fibers

**DOI:** 10.1002/fsn3.2421

**Published:** 2021-06-23

**Authors:** Pan Yang, Jinbiao Zhao

**Affiliations:** ^1^ State Key Laboratory of Animal Nutrition College of Animal Science and Technology China Agricultural University Beijing China

**Keywords:** dietary fiber, gut health, microbiota, pig, short‐chain fatty acids

## Abstract

Many studies have reported that dietary fibers play a crucial role in promoting intestinal health of the host, since it strengthens functions of epithelial barrier and meanwhile maintains intestinal homeostasis of the host by modulating gut microbiota and short‐chain fatty acid (SCFA) production. Pig is a good animal model to study effects of dietary fiber on gut health and microbial community. This review has summarized the relevant knowledge available based on roles of various dietary fibers in gut health and energy metabolism of pigs and humans. Evidences summarized in our review indicated that modulating intestinal microbial composition and SCFA production by consuming specific dietary fibers properly could be conducive to health improvement and disease prevention of the host. However, types of dietary fiber from edible foods exert divergent impacts on gut health, energy metabolism, microbial composition, and SCFA production. Therefore, more attention should be focused on different responses of various dietary fibers intake on host metabolism and health.

## Graphical abstract

Modulating intestinal microbial composition and short‐chain fatty acid (SCFA) production by regulating specific dietary fibers intake properly could be conducive to host health improvement and disease prevention. Types of dietary fiber from edible foods exert divergent impacts on gut health, microbial composition, and SCFA production.

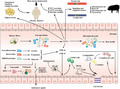

## 1 INTRODUCTION

Prebiotics are food components that can be selectively fermented, leading to changes in composition and activity of gut microbiota, then contributing to improvement of host health (Gibson et al., 2017). Dietary fibers derived from foods, which include cellulose, hemicelluloses, pectin, gums, mucilage, undigested oligosaccharide, and resistant starch, are usually considered to behave as prebiotics in human health and nutrition (Mudgil & Barak, 2013). Dietary fibers are polysaccharides which linked with more than 10 glycosidic bonds, and they are partially or completely fermented by gut microbiota in the hindgut of pigs and humans to synthesize short‐chain fatty acids (SCFA). Dietary fibers could decrease transit time of digesta, increase stool bulk, and reduce blood cholesterol and glucose (Jarrar et al., 2019; Kerckhoffs et al., 2003; Mudgil & Barak, 2013). Apart from those directly physiological responses originated from physiochemical properties of dietary fibers, it could also improve growth and activity of the intestinal microbiota, which underlie some prebiotic effects on host health and disorders prevention (Gensollen et al., 2016). Intestinal microbiota community shaped *via* microbial fermentation of dietary fiber is beneficial to host health through regulating physiological processes of the intestine and functions of mucosal immunity. More specifically, gut microbiota intensify integrity of the gut barrier comprised by intestinal epithelial cells, suppress colonization of enteric pathogens, and produce antibacterial peptides in the mucus layer of host intestine (Bäumler & Sperandio, 2016; Natividad & Verdu, 2013).

The SCFA produced by microbial fermentation of dietary fibers mainly include acetate, propionate, and butyrate, which play an important role in regulating energy metabolism, immunological function, and gut cell proliferation of the host (Koh et al., 2016). Butyrate is a source of energy for colonocytes to maintain the gut barrier, whereas acetate and propionate are delivered to peripheral circulation through the portal vein to participate in metabolisms of the liver and peripheral tissues (Liu, Wang, et al., 2018; Liu, Zhao, et al., 2018). In addition, several studies have demonstrated that SCFA has diverse metabolic and regulatory activities, such as modulating immune functions, providing energy for cell turnover, and being a histone deacetylase (HDAC) inhibitor (Flint et al., 2012; Thangaraju et al., 2009). Furthermore, there is a broad consensus that SCFA act as physiological signaling molecules to adjust biological processes associated with host health and nutrition. Many researchers reported that SCFA mediates glucose homeostasis by activating G protein‐coupled receptors (GPR 41 and 43) and stimulating enteroendocrine L‐cells to produce glucagon‐like peptide 1 (GLP‐1) and peptide YY (PYY), resulting in an increase insulin sensitivity (Mudgil & Barak, 2013; Tolhurst et al., 2012). The SCFA promotes secretion of inflammatory cytokines, such as interleukin‐6 (IL‐6), tumor necrosis factor‐α (TNF‐α), interleukin‐10 (IL‐10), and chemokine monocytes chemotactic protein‐1 (MCP‐1) to enhance intestinal immune barrier function by inhibiting activity of HDAC and stimulating expression of G protein‐coupled receptors (Montagne et al., 2003; Smith et al., 2013). Nicolucci et al. (2017) reported that obese patients who consumed inulin reduced plasma triglyceride IL‐6 concentrations. Therefore, SCFA plays a crucial role to regulate responses of dietary fiber fermentation by gut microbiota on host metabolisms and health.

As reported by Cappai et al. (2013), a higher starch digestibility from cereals is positively related to a lower amylose to amylopectin ratio in the starch composition under a same starch concentration condition, as starch with high amylose content is less digestible. Therefore, structure and composition of dietary fibers may play an important role in fiber fermentability by gut microbiota. Evidences showed effects of different types of dietary fibers derived from edible foods on gut health and energy metabolism of the host were associated with their physical characteristics and fiber composition (Zhao et al., 2019). Microbial metabolites produced from microbial fermentation of fiber are also varying when pigs and humans consume different types of dietary fiber. A higher proportion of valeric acid accounted for total SCFA was observed when hulled shredded acorns are fed to pigs (Cappai et al., 2020). Illustrating these effects on development of gastrointestinal tract in humans is challenging because of difficulty in sample collection. Alternatively, pig is a good model to study effects of dietary fibers on gut health and microbial composition in humans, considering high similarity of the intestinal biology and gut microbiota community between pigs and humans (Lee et al., 2011). Our hypothesis is that roles of different types of dietary fiber in regulating gut health and host metabolism vary. Therefore, this review summarizes effects of different dietary fibers with varying physicochemical properties derived from commercial diets on energy metabolism, gut morphology, gut barrier function, intestinal microbiota, and SCFA production in both pigs and humans, and practice of dietary intervention using dietary fibers to maintain host health and metabolism. Considering various proportions of dietary fibers derived from different fiber‐rich foods and their different physicochemical properties, it is crucial to ingest a variety of fiber‐rich foods to benefit animal and human health.

## 2 DEFINITION AND CLASSIFICATION OF DIETARY FIBERS

A widely accepted definition is that dietary fiber is one of the carbohydrates that are indigestible by endogenous enzymes in pigs and humans and meanwhile exert vital impacts on maintaining normal physiological function and energy metabolism of the host (Cummings & Stephen, 2007). Dietary fiber is mainly divided into oligosaccharides and polysaccharides. Oligosaccharides are nondigestible carbohydrates composed of 3–9 monosaccharides which are connected with either α 1–4 or α 1–6 glycosidic bonds, and mainly include fructo‐oligosaccharides, galacto‐oligosaccharides and isomalto‐oligosaccharides, human milk oligosaccharides and xylo‐oligosaccharides (Borderías et al., 2005). Edible foods provide many oligosaccharides to pigs and humans, which usually have sweet taste and exhibit prebiotic effects on gut microbiota and host health (Cheng et al., 2017). Polysaccharides are complex carbohydrates, composed of 10 up to several thousand monosaccharides, which are primarily composed of resistant starch, cellulose, hemicellulose, and β‐glucan. Cellulose is the most abundant polysaccharides consisting of up to 10,000 glucose monomer units per molecule, and it is the major component of the plant cell wall, which is only partially fermented in the intestine of pigs and humans. Hemicellulose is also a component of the plant cell wall, but it has both linear and branched molecules containing 50~200 pentose units and hexose units, such as β‐glucan, glucomannan, arabinoxylan, and xylan (Adebowale et al., 2019). β‐glucan is a branched polysaccharide with glucose polymers which lead to its high solubility and fermentability in the intestine (Bashir & Choi, 2017). Resistant starch, which is divided into four types based on its structure and fermentability, is a kind of starch that can pass through small intestine of humans without being fully digested, and reach large intestine to be degraded by beneficial gut bacteria (Sajilata et al., 2006). Pectin, characterized by high viscosity and fermentability, is composed of three different polysaccharides: homogalacturonans, rhamnogalacturonans, and galactomannans (Rejaii & Salehi, 2016). Different kinds of dietary fibers derived from edible foods are showed in Table 1. Generally, polysaccharides in legume seeds primarily consist of raffinose, stachyose, and verbascose, and cereal‐derived polysaccharides are made up of xylo‐oligosaccharide and β‐glucan. Fructo‐oligosaccharide is a main fiber component in fruits, and resistant starch is primary nondigestible carbohydrates in root vegetables. Chemical compositions and physical properties of the common cereal and cereal by‐products are presented in Tables 2 and 3, respectively. Overall, there is large variation in composition of chemical constituents and physical properties among cereal and cereal by‐products.

**TABLE 1:** Main compounds and sources of dietary fibers in foods

Categories	Degree of polymerization	Type	Structure	Source
Oligosaccharides	3–9			
	Malto‐Oligosaccharides	α‐glucans	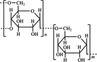	Starch hydrolysis process
	Non‐α‐glucan oligosaccharides	Raffinose	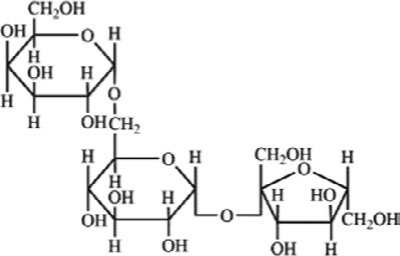	Soybean meal, peas, rapeseed meal, sunflower meal, cottonseed meal
		Stachyose	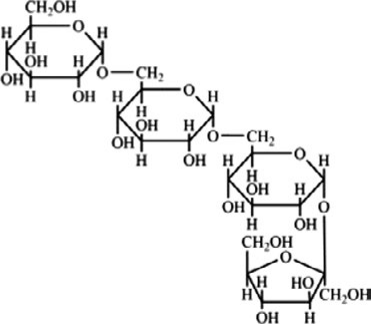	
		Verbascose	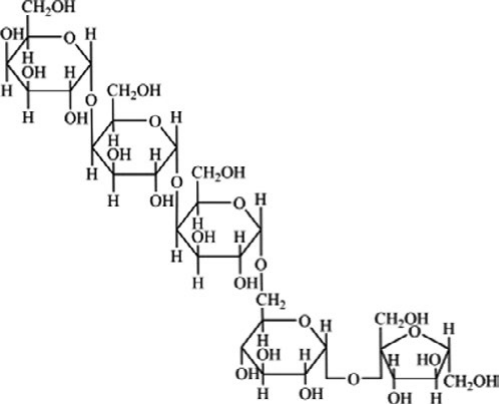	
		Fructo‐oligosaccharides	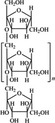	Banana, onion, barley
		Galacto‐oligosaccharides	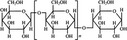	Milk
		Xylo‐oligosaccharides	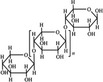	Cereals Bardana, onion
Polysaccharides	≥10			
	Resistant starch	RS1, RS2, RS3, RS4	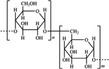	Peas, fava beans, raw potato
	Cell wall	Cellulose	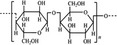	Most foods
		Mixed linked β‐glucan	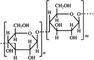	Barley, oats, rye
		Arabinoxylan	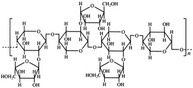	Rye, wheat, barley, cereal by‐products
		Xyloglucans	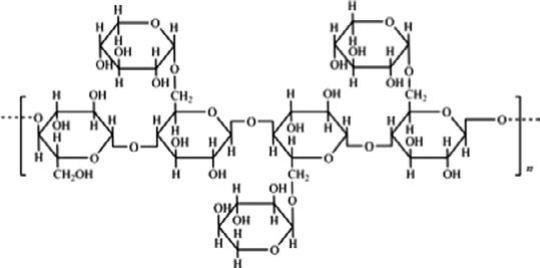	Pea hulls
		Rhamnogalacturans	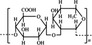	Soybean meal, sugar beet pulp
	Noncell wall	Galactans	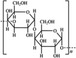	Lupins
		Fructans	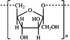	Chicory roots, rye
		Mannan	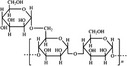	Coconut cake, palm cake
		Pectin	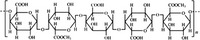	Fruits
		Guar gums	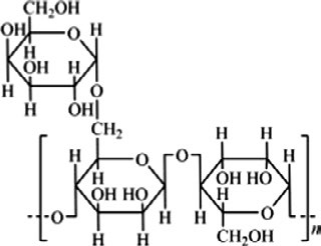	Guar

**TABLE 2:** Compositions of dietary fibers in common cereals (on dry matter basis)

Item, g/kg	Maize	Wheat	Rye	Barley	Oat
Soluble NSP	9	25	42	56	40
Rhamnose	0	0	0	0	0
Arabinose	3	7	12	6	3
Xylose	2	9	20	6	2
Mannose	2	2	2	2	2
Galactose	1	2	1	1	2
Glucose	1	4	6	39	28
Uronic acids	1	1	1	2	3
Insoluble NSP	66	74	94	88	110
Rhamnose	0	0	0	0	0
Arabinose	19	22	24	22	15
Xylose	28	38	41	50	78
Mannose	1	1	3	2	1
Galactose	4	2	4	2	5
Glucose	9	7	20	8	5
Uronic acids	6	4	3	4	7
Cellulose	22	20	16	43	82

Abbreviation: NSP, nonstarch polysaccharides.

**TABLE 3:** The compositions of dietary fibers in common cereal by‐products (on dry matter basis)

Item, g/kg	Maize			Wheat			Rye		Barley		Oat	
	Flour	Bran	Gluten	Flour	Bran	Middlings	Bran	Middlings	Dehulled	Hull meal	Feed meal	Hull meal
Soluble NSP	8	32	6	16	29	71	63	62	50	20	42	13
Rhamnose	0	0	0	0	0	0	0	0	0	0	0	0
Arabinose	3	6	1	3	7	21	11	17	4	3	2	2
Xylose	3	5	1	7	10	31	33	30	7	0	1	0
Mannose	1	1	3	0	1	2	1	1	1	0	2	1
Galactose	1	2	0	2	2	3	2	2	2	1	2	0
Glucose	0	6	2	2	8	11	13	10	34	13	33	8
Uronic acids	1	12	0	2	2	3	2	3	1	3	2	1
Insoluble NSP	13	240	14	17	273	101	321	199	58	267	39	295
Rhamnose	0	0	0	0	0	0	0	0	0	0	0	0
Arabinose	3	66	3	6	83	27	67	53	17	48	8	26
Xylose	3	111	3	8	138	36	180	89	29	184	15	212
Mannose	0	3	0	1	4	6	2	6	2	3	1	1
Galactose	0	18	0	0	7	4	10	7	0	5	1	9
Glucose	5	10	6	4	27	21	53	40	8	12	10	12
Uronic acids	2	32	2	0	13	7	8	5	2	15	3	35
Cellulose	0	83	5	3	72	19	39	27	19	192	8	196

Abbreviation: NSP, nonstarch polysaccharides.

## 3 PHYSICAL CHARACTERISTICS OF DIETARY FIBERS

Major physical characteristics of dietary fibers include the following aspects: solubility, water‐holding capacity, viscosity, swelling capacity, and bulk density. Based on solubility, dietary fibers in edible foods are divided into two categories: soluble and insoluble chemical components (Ferrario et al., 2017). Water‐holding capacity is ability of dietary fibers to combine with water for forming colloidal suspensions, and this ability depends on types of glycosidic bonds and compositions of polysaccharides (Lan et al., 2012). Viscosity is an important physical characteristic that affects physiological function of dietary fibers. Viscosity of pectin and glucan is greater than that of the cellulose and lignin in edible foods for pigs and humans (Dikeman & Fahey, 2006). Moreover, dietary fibers with long chains are easier to form net structures than short‐chain fractions, leading to greater viscosity of long‐chain dietary fibers. Swelling occurs when structure of dietary fibers solubilizes and is dispersed by incoming water, and therefore, swelling degree is dependent on water‐binding capacity of dietary fiber (Knudsen et al., 2013). Expansion and dispersion of dietary fibers allow more rapid access by microbial enzymes, resulting in increased fiber fermentability and SCFA production. Bulk density is defined as the degree of consistency measured by the quantity of mass per unit volume occupied by the fibrous materials (Elleuch et al., 2011). A lower bulk density would lead to more fullness in the gastrointestinal tract, resulting in a reduced appetite and feed intake. In the future, there will be less variation on physical characteristics of different dietary fibers, but the relationship between their physical characteristics and host health and metabolism have been barely studied.

## 4 DIETARY FIBERS AND GUT HEALTH AND ENERGY METABOLISMS OF THE HOST

**FIGURE 1:**
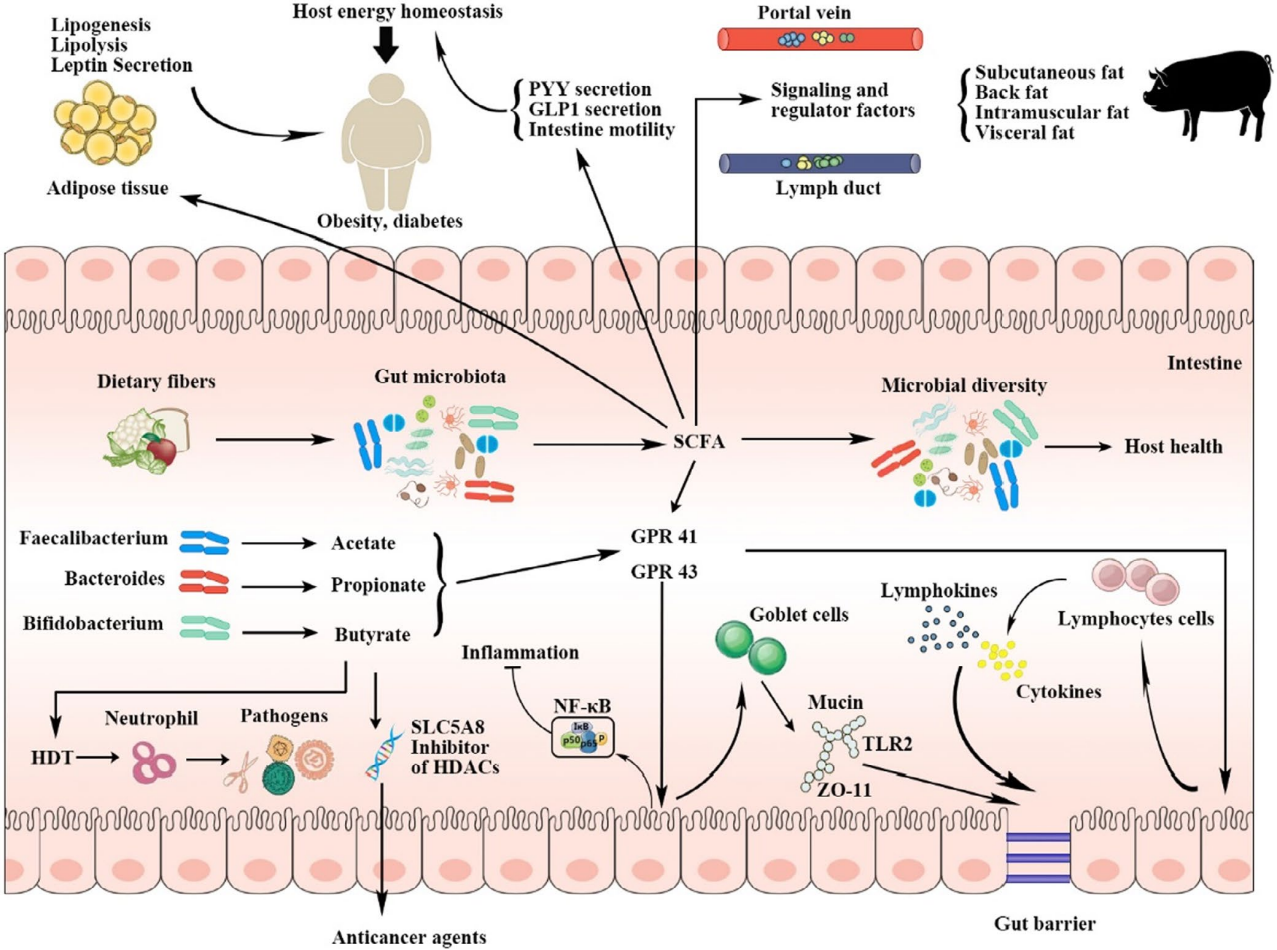
Trophic mechanisms of dietary fibers fermented by gut microbiota on energy metabolism and gut health in pigs and humans. GLP‐1, glucagon‐like peptide‐1; GPR, G protein‐coupled receptors; HDAC, histone deacetylase; HDP, human defense peptides; NF‐κB, nuclear factor‐κB; PYY, peptide YY; SCFA, short‐chain fatty acids; TLR2, Toll‐like receptor

Increasingly, researchers have proven that dietary fibers improve gut health of the host (Figure 1 and Table 4). One reason for improvement in gut health is direct responses of dietary fibers on gut development and intestinal motility, resulting in improving gut morphology and capacity of nutrient absorption. Additionally, dietary fibers shape gut microbial compositions to regulate gut health of the host. Dietary fibers are fermented by gut bacteria to produce SCFA, such as acetate, propionate, and butyrate, which enhance gut health and immune function in pigs and humans. However, different results have been reported focusing on effects of different types of dietary fibers on SCFA production, gut microbiota composition, as well as gut health and disease of the host.

**TABLE 4:** Summary of dietary interventions with dietary fibers to regulate gut health and alleviate metabolic syndrome in pig model and humans

Subject of study	Types of dietary fibers	Prebiotic response	References
Pig	Wheat, barley	Colon weight	Serena et al. (2008)
Pig	Sunflower meal, sugar beet pulp, wheat fiber	Intestine weight, amylase activity	Bikker et al. (2006)
Pig	Pearl barley	Colon weight	Hopwood et al. (2004)
Pig	Sugar beet pulp	Gut morphology	Schiavon et al. (2004)
Pig	Corn bran, wheat bran	Enzyme activity	Chen et al. (2014)
Pig	Wheat bran, pea fiber	Tight junction protein and toll‐like receptor expression	Chen et al. (2013)
Pig	Pea fiber	Colonic mucin level	Che et al. (2014)
Pig	Arabinoxylan	Goblet cell number and sIgA secretion	Chen et al. (2015)
Pig	Chicory fiber	HSP27 expression	Liu et al. (2012)
Pig	Resistant starch	Mucin secretion	Zhou et al. (2016)
Pig	Corn bran, wheat bran	Mucin‐2 expression	Vila (2017)
Hypercholesterolemic patient	β‐glucan from oat bran	Serum cholesterol	Kerckhoffs et al. (2003)
Healthy individual	Cereal fiber	Serum cholesterol	Jarrar et al. (2019)
Healthy individual	Resistant starch	Inulin sensitivity	Giles et al. (2019)
Younger and middle‐aged patient	Cereal fiber with low glucose index	Alleviate type Ⅱ diabetes	Schulze et al. (2004)
Diabetic patient	Whole grain	GLP‐1 expression, hemoglobin A1c levels	Zhao et al. (2018)
Diabetic patient	Whole grain	Plasma glucose, insulin, and ghrelin responses	Silva et al. (2015)
Healthy individual	Whole grain	Glycemic control	Venn and Mann (2004)
Healthy individual	Barley Kernel‐based bread	Gut hormones and insulin sensitivity index	Nilsson et al. (2015)
Nonobese healthy individual	Oligofructose	Hormones to regulate appetite	Pedersen et al. (2013)
Nonobese healthy individual	Fruits and vegetables	Limit long‐term weight gain	Mozaffarian et al. (2011)
Obese patient	Inulin	Serum triglyceride and IL‐6	Nicolucci et al. (2017)

Abbreviations: CRC, colorectal cancer; GLP‐1, glucagon‐like peptide‐1; HSP, heat shock protein; IL‐6, interleukin‐6.

### 4.1 Dietary fibers and intestinal development

Intake of dietary fibers reduces energy density in edible foods. To compensate, small intestines of pigs and humans need to expand area intestinal villi for improved nutrient absorption, which stimulates intestinal development to meet the nutrient requirements of the host, leading to the increased gastrointestinal tract weight (Serena et al., 2008). Physiological basis for intestinal digestion and nutrient absorption is morphology of the intestinal mucosa, including villus height and the crypt depth. Mucosa morphology reflects intestinal capacity of nutrient absorptions. A decreased ratio of villus height to crypt depth usually relates to impaired digestion and absorption of nutrients by intestinal mucosa. On the contrary, increased ratio of villus height to crypt depth usually indicates improved intestinal mucosal function, and enhanced digestion and absorption of nutrients (Furuse, 2010). Serena et al. (2008) used gestating sows to demonstrate that diets based on wheat and barley with high concentrations of dietary fibers increased colon weight of gestation pig. Bikker et al. (2006) observed that neonatal piglets fed high concentration of soluble dietary fibers sourced from wheat middlings, sunflower meal, and sugar beet pulp tended to increase length of small intestine and improve amylase activity in the small intestinal brush border. Hopwood et al. (2004) also found that pearl barley, rich in glucan and resistant starch, increased weight of colon and caecum in neonatal piglets. Similarly, dietary fibers intake increased villus height to crypt depth ratio and improved absorptive ability of the small intestine in pigs (Jha et al., 2019). Schiavon et al. (2004) showed that an improvement in viscosity of intestinal digesta accelerated cell exfoliation in the apical part of the intestinal villus by fiber intake, resulting in reduced villus height and deep crypt depth. However, intake of dietary fiber derived from corn bran and wheat bran did not affect villus height and crypt depth of the jejunum and ileum in pigs, but enhanced activities of sucrase and maltase (Chen et al., 2014). One important reason that dietary fibers can benefit development of the gastrointestinal tract is that they directly disrupt surface structure of the mucosal layer and increase speed of cell shedding, which causes compensatory growth of mucosal cells. In addition, SCFA produced by gut microbiota through microbial fermentation of dietary fibers in the large intestine of the host reduces pH of the gut, and stimulates cell division and cell proliferation. Specifically, butyrate provides energy for proliferation of the epithelial cells in the host intestine, modifies gene expression for epidermal growth factor, and repairs damaged epithelial cells, which all promote intestinal growth and development (Koh et al., 2016; Liu, Wang, et al., 2018; Liu, Zhao, et al., 2018). Furthermore, SCFA simulates secretion of gastrin and glucagon‐like peptides, which boost proliferation of the epithelial cells in the host intestine (Tolhurst et al., 2012). Overall, dietary fiber accelerates motility and development of gastrointestinal tract through its physical function and prebiotic responses of SCFA produced by microbial fermentation of dietary fiber.

### 4.2 Dietary fibers and intestinal mucosal barrier

Mucosal barrier of intestine is composed of the epithelial cell barrier and the mucosal barrier attached to the epithelial cells. Mucosal barrier mainly consists of mucins, intestinal trefoil peptide, antimicrobial peptide, cytokines, and secretory immunoglobulin A (Bai et al., 2010). Zhou et al. (2016) showed that resistant starch intake significantly increased secretion of mucin by colonic goblet cells of pigs. Vila (2017) observed that intake of cereal foods, corn bran, and wheat bran significantly improved mucin‐2 level in the ileum and colon of pigs. Che et al. (2014) demonstrated that colonic mucin level in pigs fed pea fiber diet was 16% higher than that fed a control diet. Chen et al. (2013) reported that diets supplied with 10% wheat bran or pea fiber improved gut barrier functions of the neonatal piglets, which was caused by enhanced expression of the tight junction protein between the ileum and colonic epithelial cells (ZO‐11) and the Toll‐like receptor (TLR2) mRNA (Chen et al., 2013). However, intake of corn bran and soy fiber did not affect intestinal barrier functions compared with a control group. Addition of arabinoxylan in neonatal piglet's diets significantly increased an amount of IgA secreted in the intestine and a number of goblet cells, and reduced intestinal permeability (Chen et al., 2015). Under the condition of high temperature, pigs and humans can synthesize heat shock protein (HSP) to alleviate heat stress and support stability of the mucosal barrier in the host intestine. A recent study illustrated that chicory fiber intake significantly increased expression of HSP27 in the ileum and colon of pigs, and expression of HSP27 in the ileum was positively correlated with the soluble uronic acid intake (Liu et al., 2012). Furthermore, many researchers have reported that SCFA production is beneficial to secretion of mucosal proteins in pig intestine, but concentrations and types of SCFA can influence expression of mucosal proteins (Barcelo et al., 2000; Hatayama et al., 2007). Fundamentally, high levels of dietary fibers supplementation can decrease energy density in diets, leading to an increase in food intake and greater digesta flow in the intestine, which promotes renewal of mucosal protein, thus affecting intestinal mucosal layer. Moreover, dietary fibers stimulate intestinal epithelial cells to secret mucosal protein, and produce growth factors and metabolites such as arachidonic acid, all of which are beneficial to goblet cell proliferation and mucosal protein secretion. Furthermore, dietary fibers modify intestinal barrier by altering microbial community. Desai et al. (2016) reported that there was an interactive relationship between dietary fibers and mucosal barrier of the colon. With inadequate amount of dietary fibers, gut bacteria could only maintain their own growth by using colonic mucin as a nutrient source, inevitably leading to the erosion of the mucosal barrier. It was suggested that gut bacteria play a key role in interactive relationships between dietary fibers and mucosal layer or intestinal epithelium. However, different dietary fibers have discrete structures, such as monosaccharide type, glycosidic bond, and physicochemical property, thus exerting different impacts on intestinal barrier in pigs and humans (Hamakerb & Tuncil, 2014).

### 4.3 Dietary fibers and short‐chain fatty acids production

Dietary fiber fermented by gut microbiota leads to the production of SCFA, including acetate, propionate, and butyrate, along with lactate and some gases like hydrogen, carbon dioxide, and methane (den Besten et al., 2013). Process of microbial fermentation of dietary fiber to produce SCFA involves a series of principle reactions, and is mediated by compositions and abundances of gut microbiota (Koh et al., 2016; Louis et al., 2014). Acetate accounts for about 60% of the total SCFA produced in the large intestine, whereas small quantities of propionate and butyrate are produced by microbial fermentation (Lunn & Buttriss, 2010). Lactate is primarily produced in the upper gut, but acetate, propionate, and butyrate are synthesized in the colon and cecum, which are associated with specific microbial community localization in the foregut and hindgut of the host (Zhao et al., 2019). In the foregut, *Lactobacillus* is primary lactate‐producing bacteria, and an abundance of *Lactobacillus* would reduce in the hindgut due to gut environment, such as pH and oxygen concentration. In addition, *Firmicutes* and *Bacteroides* are dominant bacteria in the hindgut and account for more than 90% of total bacteria, and *Prevotellaceae*, *Ruminococcaceae*, and *Lachnospiraceae* are primarily bacteria on the family level to produce SCFA by microbial fermentation of dietary fiber (Liu et al., 2017). The SCFA can be absorbed rapidly by intestinal epithelial cells and influence gene expression, cell differentiation, and proliferation (Mu et al., 2017). Acetate is absorbed by the portal vein and acts on energy source to muscle tissues while propionate is converted to glucose in the liver (Makki et al., 2018; Williams et al., 2001). Butyrate is easily metabolized by β‐oxidation in the mitochondria and provides from 60% to 70% of the total energy demand of colonic epithelial cells (Corrêa‐Oliveira et al., 2016; Mentschel & Claus, 2003). In addition to being an important respiratory fuel, butyrate is considered beneficial for gut health of the host because it promotes proliferation of mucosa, differentiation of epithelial cells, and function of colonic barrier in the host intestine (Mu et al., 2017).

The SCFA facilitates microbial growth and bacteriocin secretion in the intestine, and then enhances immune barrier and microbial community structure of the intestine, resulting in improving gut health of the host (Liu, Wang, et al., 2018; Liu, Zhao, et al., 2018). During process of microbial fermentation to dietary fibers, SCFA produced decreases pH of the intestinal environment and promotes proliferation of the intestinal epithelial cells (Morrison & Preston, 2016). A decreased pH provides a suitable growth environment for the beneficial bacteria, such as *Bifidobacterium* and *Lactobacillus*, and further reduces intestinal pH value and bacteria susceptible to acidic conditions, resulting in inhibiting growth of harmful bacteria and invasion of pathogens. In addition, many beneficial bacteria shaped by SCFA can secret bacteriocin to kill harmful bacteria and then improve health of the host. Bacteriocins lacticin, nisin, and bioengineered nisin variants, which are bacteriocins produced by strains of Lactococcus lactis, have been shown to be effective in vitro against clinically relevant diseases and disorders (Rea et al., 2013). The SCFA produced by microbial fermentation decreases secretion of proinflammatory cytokines, such as interleukin‐6 (IL‐6) and tumor necrosis factor‐α (TNF‐α), and promotes anti‐inflammatory cytokine interleukin‐10 (IL‐10) and chemokine monocytes chemotactic protein‐1 (MCP‐1), to enhance intestinal immune barrier function (Montagne et al., 2003). The SCFA production was also reported to modulate the immune function of the host by inhibiting the activity of HDAC and stimulating the expression of G protein‐coupled receptors (Smith et al., 2013). Sodium butyrate regulates release of interleukin‐2 (IL‐2), IL‐6, interleukin‐8 (IL‐8), and TNF‐α by inhibiting HDAC activity and activating the activator protein 1 (AP‐1) signaling pathway in intestinal epithelial cells to enhance the intestinal immune function of the host (Cox et al., 2009; Tan et al., 2014). At the same time, sodium butyrate effectively regulates function of T lymphocytes through motivating G protein‐coupled receptors (GPR43) to reduce the level of inflammatory factor IL‐2 and to increase the secretion of anti‐inflammatory factor interleukin‐4 (IL‐4) and antimicrobial peptide LL‐37, which ultimately inhibits the inflammation response of the host (Cleophas et al., 2016; Macpherson et al., 2008). Overall, these results indicate that dietary fiber plays a crucial part in immune function of the host by increasing the SCFA concentration.

Many researchers reported that SCFA mediates glucose homeostasis and fat acids metabolism in the host by activating GPR41, GPR43, and stimulating enteroendocrine L‐cells to produce GLP‐1 and peptide YY, resulting in improved insulin sensitivity (Mudgil & Barak, 2013; Tolhurst et al., 2012). Pedersen et al. (2013) reported that oligofructose stimulated GLP‐1 and insulin secretion to increase host appetite, resulting in depressing intake of food and incidence of obesity. Furthermore, a consumption of barley kernel‐based bread to healthy human volunteers, which is rich in β‐glucan, improved glucose metabolism and prevented the risk of obese disease (Nilsson et al., 2015). Similar, Mozaffarian et al. (2011) reported that nonobese healthy individual who consumed fibers from fruits and vegetables could limit weight gain for a long time. In addition, a recent study assessed the relationship between various types of dietary carbohydrates and insulin resistance, and results showed adolescents with dietary fiber intervention reduced possibility of insulin resistance, but no significantly associations were observed for rest of the carbohydrate variables (Castro‐Quezada et al., 2019). Resistant starch consumption decreased digestible carbohydrates oxidation and serum glucose concentration with improved insulin sensitivity after meals (Giles et al., 2019). Therefore, it is a promising strategy to regulate glucose and fatty acids metabolism, and prevent human obesity and diabetes by specific dietary fiber consumption by modulating cellular receptors of GPRs (Schulze et al., 2004; Venn & Mann, 2004). However, Haenen et al. (2013) did not find an improvement in intestinal expression of GPR41 and GPR43 when pigs were fed diets high in resistant starch. Nielsen et al. (2015) observed a lower expression of GPR41 when feeding a diet supplemented with high concentration of arabinoxylan. Furthermore, Hooda et al. (2010) reported that oat β‐glucan intake increased the net SCFA absorption in the portal vein of catheterized pigs, but reduced the production of insulin mediated by GLP‐1 activity, which is consistent with the previous finding in diabetic patients (Silva et al., 2015). Ingerslev et al. (2014) did not observe a link between SCFA absorption and the portal GLP‐1 flux. Discrepancy of observations above should be primarily associated with types and structures of dietary fibers.

Among the dietary fiber fractions, β‐glucan is receiving more attention because it is an easily fermentable energy source for intestinal microbiota. β‐glucan is certainly fermented by most of gut microbiota, except for *Enterobacteriaceae* (Stack et al., 2010). Intestinal microbiota could produce lactic acid to reduce intestinal pH and further selectively facilitate proliferation of *Lactobacillus* and *Bifidobacterium* (Stack et al., 2010). Oat bran, a soluble dietary fiber source rich in β‐glycan, produces almost twice the amount of SCFA per gram of dietary fiber compared with wheat bran during microbial fermentation (Zhao et al., 2020). Bach Knudsen and Canibe (2000) detected higher concentration of lactate (11.6 vs. 3.4 mmol/ kg of digested feed) and greater proportion of butyrate accounted for total organic acid (9.1% vs. 6.1%) in the small intestine of pig's model after feeding diets supplemented with oat bran than wheat bran. Zhao et al. (2019) reported that intake of oat bran by pigs had significantly distinct improvement on amounts of lactic acid produced in the foregut, and soybean hulls and sugar beet pulp fed to pigs. They also observed acetate, propionate, and butyrate concentrations in the hindgut in oral bran treatment were higher than corn bran and wheat bran. Freire et al. (2000) investigated the effects of dietary wheat bran, sugar beet pulp, soybean hulls, or alfalfa meal intake on total SCFA production in the cecum of pigs. They found that dietary soybean hulls consumption increased total SCFA concentration by 11.2%, 30.5%, and 27.2% compared with dietary wheat bran, sugar beet pulp, and alfalfa consumption, respectively (Freire et al., 2000). Overall, variation in fermentability and SCFA production among different types of dietary fibers could be mainly ascribed to the differences in their chemical compositions and physicochemical properties. There is great potential to improve gut health and immune function of humans by regulating the intake of different kinds of dietary fibers to manipulate the production of SCFA.

The present argument is that SCFA produced in the intestine not only derived from microbial fermentation of dietary fibers, but also resulted from the secretion of the nondietary fiber components (Montoya et al., 2016). Montoya et al. (2017) reported that SCFA sourced from in vitro fermentation of dietary fibers in kiwifruit using fecal microbiota of humans only accounted for 30% of the total SCFA production. They explained that the main endogenous nondietary fiber components are soluble dietary fibers derived from the intestinal mucin in the small intestine, whereas microbial cell was the main component of the main endogenously losses as an insoluble dietary fiber in the whole gastrointestinal tract (Montoya et al., 2017).

In addition, the SCFA concentration measured in many in vivo studies only came from microbial fermentation, but a part of SCFA absorption by epithelial cells in the gut of the host was usually ignored. A common method to quantify the net absorption of SCFA is to collect blood samples from the portal vein and mesenteric artery simultaneously, and then analyze the SCFA concentrations via a portal vein‐catheterized pig model. Net portal absorption and concentration of SCFA in the portal vein or mesenteric artery estimated via catheterized pigs depend on the types of dietary fibers in cereal‐based diets fed to pigs. For instance, diets rich in arabinoxylan can stimulate proliferation of butyrate‐producing microorganisms and butyrate production in the large intestine, and increase the net portal absorption of butyrate compared with diets high in resistant starch with equal amount of dietary fibers (Ingerslev et al., 2014; Nielsen et al., 2015). Therefore, it would be extremely difficult to measure actual produced SCFA in vivo, as produced SCFA are rapidly metabolized by gut microbiota or absorbed by the host. The absorption and net production of SCFA, rather than a real‐time concentration of SCFA in the gut, derived from gut microbiota to ferment different types of dietary fibers should be quantified to understand the fermentable capacity of dietary fibers and the metabolic pathway of SCFA in the gut of the host.

### 4.4 Dietary fibers and gut microbiota

Microbial community in the host intestine is a complex and dynamic ecosystem that produces crucial metabolites to regulate host metabolism, such as SCFA, 5‐hydroxytryptamine, polymyxin, and bacitracin. Microbial metabolites depress proliferation of harmful bacteria and balance interactive competition between “beneficial bacteria” and “harmful bacteria.” In addition, microbial metabolites play important roles in maintaining intestinal barrier, facilitating immunological function, and modulating gene expression of host metabolism (Cani, 2016; Guo et al., 2008). An increment of microbial activity was found in the intestine of pigs fed diets containing a high content of dietary fiber, as indicated by increased bacterial counts and ATP concentration (Liu, Wang, et al., 2018; Liu, Zhao, et al., 2018). It indicates that dietary fibers can activate microbial activity, resulting in producing more microbial metabolites. Many reports have indicated that *Firmicutes* and *Bacteroidetes* are the two dominant phyla in the gastrointestinal tract of pigs and humans, which account for about 90% of the gut microbiota (Bian et al., 2016; Liu, Wang, et al., 2018; Liu, Zhao, et al., 2018). *Firmicutes* utilize dietary fibers to produce SCFA, especially butyrate. *Bacteroidetes* have a great capacity for degradation of dietary fibers to produce propionate, such as *Prevotella* (Flint et al., 2008). Mu et al. (2017) reported that dietary supplementation with alfalfa meal increased populations of *Firmicutes* and *Bacteroidetes* compared to wheat bran in neonatal piglets. Similarly, diets rich in resistant starch increased relative populations of some specific members of Firmicutes, as well as a ratio of *Firmicutes* to *Bacteroidetes* in human's intestine (Maier et al., 2017). In contrast, Ferrario et al. (2017) detected an increase in numbers of *Bacteroidetes* and a decrease in populations of *Firmicutes* in subjects supplied with dietary inulin. Further, specific bacteria in phylum of *Firmicutes* and *Bacteroidetes* to ferment different types of dietary fibers has not been known well.

A population of *Lachnoclostridium* in fecal samples is influenced by dietary fibers supplementation, which has a connection with the obesity in humans (Amadou et al., 2016). The abundance of *Ruminococcus_1* increased when pigs were fed diets containing soybean hulls, and it was reported that *Ruminococcus_1* can ferment dietary fibers to produce SCFA (Pryde et al., 2002). Che et al. (2014) and Chen et al. (2014) showed that diets containing pea fiber increased the number of *Lactobacillus* in the colon of pigs, but pea fiber had no significant effects on the number of *Bifidobacteria* and *Escherichia coli*, while diets containing wheat bran significantly increased the number of *Bifidobacteria* in pigs. The relative abundances of *Lactobacillus*, the dominant species of the lactate‐producing bacteria in the ileum, and *Prevotella* in the colon were positively correlated with the concentration of dietary chicory fiber (Liu et al., 2012). Furthermore, high resistant starch supplementation in diets of adult pigs increased the relative abundances of *Faecalibacterium prausnitzii* and *Ruminococcus bromii*, and reduced the population of pathogenic bacteria *Escherichia coli* and *Pseudomonas* (Haenen et al., 2013).

Improved numbers of *Bifidobacteria* spp. and a decrease populations of total anaerobes or *Clostridia* in the feces were observed when the pigs were fed diets containing 0.5% fructo‐oligosaccharide in combination with *Bifidobacterium* longum (Clarke et al., 2017). Zhao et al. (2018) reported that dietary barley supplementation increased *Lactobacilli* spp. and *Bifidobacterium* spp. populations, but decreased numbers of *Enterobacteria* spp. in the cecum of pigs in comparison with a corn‐based diet, which may be attributed to greater β‐glucan levels in barley diet compared with a corn diet (Zhao et al., 2018). Oat fiber and β‐glucan isolates, coming from fermented oat‐based products containing both native and microbial β‐glucan, promote growth of *Bifidobacteria* spp. in the intestine of pigs and humans (Martensson et al., 2005). Pieper et al. (2008) found that dehulling barley with high amounts of soluble nonstarch polysaccharides favored growth of xylan‐degarding and glucan‐degrading bacteria (Pieper et al., 2008), whereas β‐glucan from hulled barleys favored growth of *Lactobacilli* spp. in weaned pigs (Pieper et al., 2008). These results suggest that different sources of β‐glucan have inconsistent responses on microbial composition and abundance in the intestine of the host, which could be associated with the structure and physical properties of different β‐glucan source, such as solubility and molecule weight. Moreover, an increase of *Bifidobacteria* spp. and *Enterobacteria* spp. abundances in ileal digesta were observed when growing pigs were fed diets supplemented with guar gum or cellulose (Owusu‐Asiedu et al., 2006). Nielsen et al. (2014) showed that diets supplemented with arabinoxylan increased populations of *Bifidobacterium* spp. and *Lactobacillus* spp. in the colon of pigs.

Small intestine is mainly responsible for food digestion and absorption, while large intestine is important for microbial fermentation of substances (Healey et al., 2020). Hindgut of pigs and humans contain a larger proportion of *Firmicutes* than small intestine, indicating that large intestine might undertake a crucial role in microbial fermentation of dietary fiber (Flint et al., 2008). *Escherichia‐Shigella*, *Lactobacillus*, *Streptococcus,* and *Enterococcus*, dominant genera of intestinal microbiota in ileal digesta, are the major bacterial species with greater abundances compared with large intestine of the pig (Zhao et al., 2019). Greater populations of *Escherichia‐Shigella* and *Streptococcus* are always considered as pathogenic bacteria related to host infection and enteric diseases, such as diarrhea symptoms. Thus, occurrences of enteropathies are primarily associated with microbial composition in the upper gut (Healey et al., 2020). *Lactobacillus* is a beneficial bacterial specie to improve gut health of the host, so that it is extremely crucial to maintain balance of different bacteria in the gut by dietary nutrients intervention. An altered gut microbiota composition derived from lack of low dietary fibers could lead to a severe deterioration of mucus layer and increased susceptibility to infections and chronic inflammatory diseases (Desai et al., 2016; Makki et al., 2018). Therefore, dietary fiber ingested from foods has a potential to prevent against metabolic diseases in humans by reshaping composition of gut microbiota. However, effects of dietary fiber on intestinal bacterial community vary among different studies, which could be attributed to different types of dietary fiber types, as well as variation on quantity of dietary fiber, available for microbial fermentation in the gut. Furthermore, a study reported consuming inulin at breakfast after a longer fasting period had a greater response on fecal microbiota of individuals (Sasaki et al., 2019), which indicated dietary habit play an important role in regulation of microbial community after ingesting dietary fibers.

## 5 SUMMARY AND PERSPECTIVE

There are many complicated and subtle interactions between types of dietary fiber and gut microbiota, SCFA production, and host health. It has been widely accepted that SCFA, especially butyrate, plays a critical role in modulating and improving gut health. However, there are still many vague aspects about underlying relationships between dietary fiber and host health. Major challenges related to dietary fiber research may include (1) to identify the specific mechanisms of SCFA produced from dietary fiber fermented by gut microbiota on functions of animal tissues and organs, (2) to clarify the relationship between host health and gut microbiota shaped by the dietary intervention with dietary fibers, (3) to further illustrate different responses of various dietary fibers derived from edible foods and their combinations on host health. Overall, we suggest that more attention should be focused on specific chemical constituents and physical characteristics of various dietary fibers when they take actions in regulating host metabolism and health.

## CONFLICT OF INTEREST

None of the authors had a financial or personal conflict of interest in relation to the present study.

## AUTHOR CONTRIBUTIONS

**Pan Yang:** Conceptualization (supporting); Data curation (supporting); Formal analysis (lead); Investigation (lead); Visualization (lead); Writing‐original draft (lead). **Jinbiao Zhao:** Conceptualization (lead); Data curation (lead); Formal analysis (supporting); Funding acquisition (lead); Project administration (lead); Writing‐review & editing (lead).

## ETHICAL APPROVAL AND CONSENT TO PARTICIPATE

Not applicable.

### DATA AVAILABILITY STATEMENT

All authors consent that raw data presented in this review are available after publication. Please contact author for data requests.

## References

[fsn32421-bib-0001] Adebowale, T. O., Yao, K., & Oso, A. O. (2019). Major cereal carbohydrates in relation to intestinal health of monogastric animals: A review. Animal Nutrition, 5, 331–339. 10.1016/j.aninu.2019.09.001 31890909 PMC6920401

[fsn32421-bib-0002] Amadou, T., Hosny, M., La Scola, B., & Cassir, N. (2016). “Lachnoclostridium bouchesdurhonense,” a new bacterial species isolated from human gut microbiota. New Microbes and New Infections, 13, 69–70. 10.1016/j.nmni.2016.06.015 27493758 PMC4963250

[fsn32421-bib-0003] Bach Knudsen, K. E., & Canibe, N. (2000). Breakdown of plant carbohydrates in the digestive tract of pigs fed on wheat‐ or oat‐based rolls. Journal of the Science of Food and Agriculture, 80, 1253–1261. 10.1002/1097-0010(200006)80:8<1253:AID-JSFA632>3.0.CO;2-0

[fsn32421-bib-0004] Bai, Z., Zhang, Z., Ye, Y., & Wang, S. (2010). Sodium butyrate induces differentiation of gastric cancer cells to intestinal cells via the PTEN/phosphoinositide 3‐kinase pathway. Cell Biology International, 34, 1141–1145. 10.1042/CBI20090481 20718712

[fsn32421-bib-0005] Barcelo, A., Claustre, J., Moro, F., Chayvialle, J. A., Cuber, J. C., & Plaisancié, P. (2000). Mucin secretion is modulated by luminal factors in the isolated vascularly perfused rat colon. Gut, 46, 218–224. 10.1136/gut.46.2.218 10644316 PMC1727811

[fsn32421-bib-0097] Bashir, K. M. I., & Choi, J. (2017). Clinical and physiological perspectives of β‐glucans: The past, present, and future. International Journal of Molecular Sciences, 18, 1906. 10.3390/ijms18091906 PMC5618555 28872611

[fsn32421-bib-0006] Bäumler, A. J., & Sperandio, V. (2016). Interactions between the microbiota and pathogenic bacteria in the gut. Nature, 535, 85–93. 10.1038/nature18849 27383983 PMC5114849

[fsn32421-bib-0007] Bian, G., Ma, S., Zhu, Z., Su, Y., Zoetendal, E. G., Mackie, R., Liu, J., Mu, C., Huang, R., Smidt, H., & Zhu, W. (2016). Age, introduction of solid feed and weaning are more important determinants of gut bacterial succession in piglets than breed and nursing mother as revealed by a reciprocal cross‐fostering model. Environmental Microbiology, 18, 1566–1577. 10.1111/1462-2920.13272 26940746

[fsn32421-bib-0008] Bikker, P., Dirkzwager, A., Fledderus, J., Trevisi, P., le Huërou‐Luron, I. , Lallès, J. P., & Awati, A. (2006). The effect of dietary protein and fermentable carbohydrates levels on growth performance and intestinal characteristics in newly weaned piglets. Journal of Animal Science, 84, 3337–3345. 10.2527/jas.2006-076 17093226

[fsn32421-bib-0009] Borderías, A. J., Sánchez‐Alonso, I., & Pérez‐Mateos, M. (2005). New applications of fibres in foods: Addition to fishery products. Trends in Food Science & Technology, 16, 458–465. 10.1016/j.tifs.2005.03.011

[fsn32421-bib-0010] Cani, P. D. (2016). Interactions between gut microbes and host cells control gut barrier and metabolism. International Journal of Obesity Supplements, 6, S28–S31. 10.1038/ijosup.2016.6 28685027 PMC5485881

[fsn32421-bib-0011] Cappai, M. G., Alesso, G. A., Nieddu, G., Sanna, M., & Pinna, W. (2013). Electron microscopy and composition of raw acorn starch in relation to in vivo starch digestibility. Food & Function, 4, 917. 10.1039/c3fo60075k 23660700

[fsn32421-bib-0012] Cappai, M. G., Wolf, P., Pinna, W., Rust, P., & Kamphues, J. (2020). Pre‐caecal disappearance of starch and volatile fatty acid (VFA) content in digesta of caecum of growing pigs fed with ripe hulled shredded acorns in their diet. Agriculture, 10, 508. 10.3390/agriculture10110508

[fsn32421-bib-0013] Castro‐Quezada, I., Flores‐Guillén, E., Núñez‐Ortega, P. E., Irecta‐Nájera, C. A., Sánchez‐Chino, X. M., Mendez‐Flores, O. G., Olivo‐Vidal, Z. E., García‐Miranda, R., Solís‐Hernández, R., & Ochoa‐Díaz‐López, H. (2019). Dietary carbohydrates and insulin resistance in adolescents from marginalized areas of Chiapas, México. Nutrients, 11(12), 3066. 10.3390/nu11123066 PMC6950049 31888175

[fsn32421-bib-0014] Che, L., Chen, H., Yu, B., He, J., Zheng, P., Mao, X., Yu, J., Huang, Z., & Chen, D. (2014). Long‐term intake of pea fiber affects colonic barrier function, bacterial and transcriptional profile in pig model. Nutrition and Cancer, 66(3), 388–399. 10.1080/01635581.2014.884229 24611475

[fsn32421-bib-0015] Chen, H., Mao, X. B., Che, L. Q., Yu, B., He, J., Yu, J., Han, G., Huang, Z., Zheng, P., & Chen, D. (2014). Impact of fiber types on gut microbiota, gut environment and gut function in fattening pigs. Animal Feed Science and Technology, 195, 101–111. 10.1016/j.anifeedsci.2014.06.002

[fsn32421-bib-0016] Chen, H., Mao, X., He, J., Yu, B., Huang, Z., Yu, J., Zheng, P., & Chen, D. (2013). Dietary fibre affects intestinal mucosal barrier function and regulates intestinal bacteria in weaning piglets. British Journal of Nutrition, 110, 1837–1848. 10.1017/S0007114513001293 23656640

[fsn32421-bib-0017] Chen, H., Mao, X., Yin, J., Yu, B., He, J., Che, L., Yu, J., Huang, Z., Zheng, P., Michiels, J., De Smet, S., & Chen, D. (2015). Comparison of jejunal digestive enzyme activities, expression of nutrient transporter genes, and apparent fecal digestibility in weaned piglets fed diets with varied sources of fiber. Journal of Animal and Feed Science, 24, 41–47. 10.22358/jafs/65651/2015

[fsn32421-bib-0018] Cheng, W., Lu, J., Li, B., Lin, W., Zhang, Z., Wei, X., Sun, C., Chi, M., Bi, W., Yang, B., Jiang, A., & Yuan, J. (2017). Effect of functional oligosaccharides and ordinary dietary fiber on intestinal microbiota diversity. Frontiers in Microbiology, 8, 1750. 10.3389/fmicb.2017.01750 28979240 PMC5611707

[fsn32421-bib-0019] Clarke, S. T., Brooks, S., Inglis, G. D., Yanke, L. J., Green, J., Petronella, N., Ramdath, D. D., Bercik, P., Green‐Johnson, J. M., & Kalmokoff, M. (2017). Impact of β2‐1 fructan on faecal community change: Results from a placebo‐controlled, randomised, double‐blinded, cross‐over study in healthy adults. The British Journal of Nutrition, 118(6), 441–453. 10.1017/S0007114517002318 28954640

[fsn32421-bib-0020] Cleophas, M. C., Crişan, T. O., Lemmers, H., Toenhake‐Dijkstra, H., Fossati, G., Jansen, T. L., Dinarello, C. A., Netea, M. G., & Joosten, L. A. (2016). Suppression of monosodium urate crystal‐induced cytokine production by butyrate is mediated by the inhibition of class I histone deacetylases. Annals of the Rheumatic Diseases, 75, 593–600. 10.1136/annrheumdis-2014-206258 25589513

[fsn32421-bib-0021] Corrêa‐Oliveira, R., Fachi, J. L., Vieira, A., Sato, F. T., & Vinolo, M. A. (2016). Regulation of immune cell function by short‐chain fatty acids. Clinical & Translational Immunology, 5, e73. 10.1038/cti.2016.17 27195116 PMC4855267

[fsn32421-bib-0022] Cox, M. A., Jackson, J., Stanton, M., Rojas‐Triana, A., Bober, L., Laverty, M., Yang, X., Zhu, F., Liu, J., Wang, S., Monsma, F., Vassileva, G., Maguire, M., Gustafson, E., Bayne, M., Chou, C. C., Lundell, D., & Jenh, C. H. (2009). Short‐chain fatty acids act as antiinflammatory mediators by regulating prostaglandin E(2) and cytokines. World Journal of Gastroenterology, 15, 5549–5557. 10.3748/wjg.15.5549 19938193 PMC2785057

[fsn32421-bib-0023] Cummings, J. H., & Stephen, A. M. (2007). Carbohydrate terminology and classification. European Journal of Clinical Nutrition, 61(1), S5–S18. 10.1038/sj.ejcn.1602936 17992187

[fsn32421-bib-0024] den Besten, G. , van Eunen, K. , Groen, A. K., Venema, K., Reijngoud, D. J., & Bakker, B. M. (2013). The role of short‐chain fatty acids in the interplay between diet, gut microbiota, and host energy metabolism. Journal of Lipid Research, 54(9), 2325–2340. 10.1194/jlr.R036012 23821742 PMC3735932

[fsn32421-bib-0025] Desai, M. S., Seekatz, A. M., Koropatkin, N. M., Kamada, N., Hickey, C. A., Wolter, M., Pudlo, N. A., Kitamoto, S., Terrapon, N., Muller, A., Young, V. B., Henrissat, B., Wilmes, P., Stappenbeck, T. S., Núñez, G., & Martens, E. C. (2016). A dietary fiber‐deprived gut microbiota degrades the colonic mucus barrier and enhances pathogen susceptibility. Cell, 167, 1339–1353.e21. 10.1016/j.cell.2016.10.043 27863247 PMC5131798

[fsn32421-bib-0026] Dikeman, C. L., & Fahey, G. C. (2006). Viscosity as related to dietary fiber: A review. Critical Reviews in Food Science and Nutrition, 46(8), 649–663. 10.1080/10408390500511862 17092830

[fsn32421-bib-0027] Elleuch, M., Bedigian, D., Roiseux, O., Besbes, S., Blecker, C., & Attia, H. (2011). Dietary fibre and fibre‐rich by‐products of food processing: Characterisation, technological functionality and commercial applications: A review. Food Chemistry, 124(2), 411–421. 10.1016/j.foodchem.2010.06.077

[fsn32421-bib-0028] Ferrario, C., Statello, R., Carnevali, L., Mancabelli, L., Milani, C., Mangifesta, M., Duranti, S., Lugli, G. A., Jimenez, B., Lodge, S., Viappiani, A., Alessandri, G., Dall'Asta, M., Del Rio, D., Sgoifo, A., van Sinderen, D. , Ventura, M., & Turroni, F. (2017). How to feed the mammalian gut microbiota: Bacterial and metabolic modulation by dietary fibers. Frontiers in Microbiology, 8, 1749. 10.3389/fmicb.2017.01749 28955319 PMC5600934

[fsn32421-bib-0029] Flint, H. J., Bayer, E. A., Rincon, M. T., Lamed, R., & White, B. A. (2008). Polysaccharide utilization by gut bacteria: Potential for new insights from genomic analysis. Nature Reviews Microbiology, 6, 121–131. 10.1038/nrmicro1817 18180751

[fsn32421-bib-0030] Flint, H. J., Scott, K. P., Louis, P., & Duncan, S. H. (2012). The role of the gut microbiota in nutrition and health. Nature Reviews Gastroenterology & Hepatology, 9(10), 577–589. 10.1038/nrgastro.2012.156 22945443

[fsn32421-bib-0031] Freire, J. P. B., Guerreiro, A. J. G., Cunha, L. F., & Aumaitre, A. (2000). Effect of dietary fibre source on total tract digestibility, caecum volatile fatty acids and digestive transit time in the weaned piglet. Animal Feed Science and Technology, 87, 71–83. 10.1016/S0377-8401(00)00183-8

[fsn32421-bib-0032] Furuse, M. (2010). Molecular basis of the core structure of tight junctions. Cold Spring Harbor Perspectives in Biology, 2, a002907. 10.1101/cshperspect.a002907 20182608 PMC2827901

[fsn32421-bib-0033] Gensollen, T., Iyer, S. S., Kasper, D. L., & Blumberg, R. S. (2016). How colonization by microbiota in early life shapes the immune system. Science, 352, 539–544. 10.1126/science.aad9378 27126036 PMC5050524

[fsn32421-bib-0034] Gibson, G. R., Hutkins, R., Sanders, M. E., Prescott, S. L., Reimer, R. A., Salminen, S. J., Scott, K., Stanton, C., Swanson, K. S., Cani, P. D., Verbeke, K., & Reid, G. (2017). Expert consensus document: The International Scientific Association for Probiotics and Prebiotics (ISAPP) consensus statement on the definition and scope of prebiotics. Nature Reviews Gastroenterology & Hepatology, 14, 491–502. 10.1038/nrgastro.2017.75 28611480

[fsn32421-bib-0035] Giles, E. D., Brown, I. L., MacLean, P. S., Pan, Z., Melanson, E. L., Heard, K. J., Cornier, M. A., Marden, T., & Higgins, J. A. (2019). The in vivo net energy content of resistant starch and its effect on macronutrient oxidation in healthy adults. Nutrients, 11, 2484. 10.3390/nu11102484 PMC6835355 31623184

[fsn32421-bib-0036] Guo, X., Xia, X., Tang, R., Zhou, J., Zhao, H., & Wang, K. (2008). Development of a real‐time PCR method for Firmicutes and Bacteroidetes in faeces and its application to quantify intestinal population of obese and lean pigs. Letters in Applied Microbiology, 47, 367–373. 10.1111/j.1472-765X.2008.02408.x 19146523

[fsn32421-bib-0037] Haenen, D., Zhang, J., Souza da Silva, C., Bosch, G., van der Meer, I. M. , van Arkel, J. , van den Borne, J. J. , Pérez Gutiérrez, O., Smidt, H., Kemp, B., Müller, M., & Hooiveld, G. J. (2013). A diet high in resistant starch modulates microbiota composition, SCFA concentrations, and gene expression in pig intestine. The Journal of Nutrition, 143, 274–283. 10.3945/jn.112.169672 23325922

[fsn32421-bib-0098] Hamakerb, R., & Tuncil, Y. E. A. (2014). Perspective on the complexity of dietary fiber structures and their potential effect on the gut microbiota. Journal of Molecular Biology, 426, 3838–3850. 10.1016/j.jmb.2014.07.028 25088686

[fsn32421-bib-0038] Hatayama, H., Iwashita, J., Kuwajima, A., & Abe, T. (2007). The short chain fatty acid, butyrate, stimulates MUC2 mucin production in the human colon cancer cell line, LS174T. Biochemical and Biophysical Research Communications, 356, 599–603. 10.1016/j.bbrc.2007.03.025 17374366

[fsn32421-bib-0039] Healey, G. R., Celiberto, L. S., Lee, S. M., & Jacobson, K. (2020). Fiber and prebiotic interventions in pediatric inflammatory bowel disease: What role does the gut microbiome play? Nutrients, 12(10), 3204. 10.3390/nu12103204 PMC7589214 33092150

[fsn32421-bib-0040] Hooda, S., Matte, J. J., Vasanthan, T., & Zijlstra, R. T. (2010). Dietary oat beta‐glucan reduces peak net glucose flux and insulin production and modulates plasma incretin in portal‐vein catheterized grower pigs. The Journal of Nutrition, 140, 1564–1569. 10.3945/jn.110.122721 20660287

[fsn32421-bib-0041] Hopwood, D. E., Pethick, D. W., Pluske, J. R., & Hampson, D. J. (2004). Addition of pearl barley to a rice‐based diet for newly weaned piglets increases the viscosity of the intestinal contents, reduces starch digestibility and exacerbates post‐weaning colibacillosis. British Journal of Nutrition, 92, 419–427. 10.1079/bjn20041206 15469645

[fsn32421-bib-0042] Ingerslev, A. K., Theil, P. K., Hedemann, M. S., Lærke, H. N., & Bach Knudsen, K. E. (2014). Resistant starch and arabinoxylan augment SCFA absorption, but affect postprandial glucose and insulin responses differently. British Journal of Nutrition, 111, 1564–1576. 10.1017/S0007114513004066 24507768

[fsn32421-bib-0043] Jarrar, A. H., Beasley, J. M., Ohuma, E. O., Ismail, L. C., Qeshta, D. A., Mohamad, M. N., & Dhaheri, A. S. (2019). Effect of high fiber cereal intake on satiety and gastrointestinal symptoms during Ramadan. Nutrients, 11, 939. 10.3390/nu11040939 PMC6521042 31027300

[fsn32421-bib-0044] Jha, R., Fouhse, J. M., Tiwari, U. P., Li, L., & Willing, B. P. (2019). Dietary fiber and intestinal health of monogastric animals. Frontiers in Veterinary Science, 6, 48. 10.3389/fvets.2019.00048 30886850 PMC6409295

[fsn32421-bib-0045] Kerckhoffs, D. A. J. M., Hornstra, G., & Mensink, R. P. (2003). Cholesterol‐lowing effect of β‐glucan from oat bran in midly hypercholesterolemic subjects may decrease when β‐glucan is incorporated into bread and cookies. American Journal of Clinical Nutrition, 78, 221–227. 10.1093/ajcn/78.2.221 12885701

[fsn32421-bib-0046] Knudsen, K. E. B., Lærke, H. N., & Jørgensen, H. (2013). Carbohydrates and carbohydrate utilization in swine. In L. I. Chiba (Ed.), Sustainable swine nutrition (pp. 109–137). Wiley‐Blackwell.

[fsn32421-bib-0047] Koh, A., De Vadder, F., Kovatcheva‐Datchary, P., & Bäckhed, F. (2016). From dietary fiber to host physiology: Short‐chain fatty acids as key bacterial metabolites. Cell, 165(6), 1332–1345. 10.1016/j.cell.2016.05.041 27259147

[fsn32421-bib-0048] Lan, G., Chen, H., Chen, S., & Tian, J. (2012). Chemical composition and physicochemical properties of dietary fiber from *Polygonatum odoratum* as affected by different processing methods. Food Research International, 49(1), 406–410. 10.1016/j.foodres.2012.07.047

[fsn32421-bib-0049] Lee, J. E., Lee, S., Sung, J., & Ko, G. (2011). Analysis of human and animal fecal microbiota for microbial source tracking. The ISME Journal, 5, 362–365. 10.1038/ismej.2010.120 20686512 PMC3105695

[fsn32421-bib-0050] Liu, H. Y., Lundh, T., Dicksved, J., & Lindberg, J. E. (2012). Expression of heat shock protein 27 in gut tissue of growing pigs fed diets without and with inclusion of chicory fiber. Journal of Animal Science, 90, 25–27. 10.2527/jas.53724 23365273

[fsn32421-bib-0051] Liu, H., Wang, J., He, T., Becker, S., Zhang, G., Li, D., & Ma, X. (2018). Butyrate: A double‐edged sword for health? Advances in Nutrition, 9, 21–29. 10.1093/advances/nmx009 29438462 PMC6333934

[fsn32421-bib-0052] Liu, P., Zhao, J., Guo, P., Lu, W., Geng, Z., Levesque, C. L., Johnston, L. J., Wang, C., Liu, L., Zhang, J., Ma, N., Qiao, S., & Ma, X. (2017). Dietary corn bran fermented by *Bacillus subtilis* MA139 decreased gut cellulolytic bacteria and microbiota diversity in finishing pigs. Frontiers in Cellular and Infection Microbiology, 7, 526. 10.3389/fcimb.2017.00526 29312900 PMC5744180

[fsn32421-bib-0053] Liu, P., Zhao, J., Wang, W., Guo, P., Lu, W., Wang, C., Liu, L., Johnston, L. J., Zhao, Y., Wu, X., Xu, C., Zhang, J., & Ma, X. (2018). Dietary corn bran altered the diversity of microbial communities and cytokine production in weaned pigs. Frontiers in Microbiology, 9, 2090. 10.3389/fmicb.2018.02090 30233555 PMC6131307

[fsn32421-bib-0054] Louis, P., Hold, G. L., & Flint, H. J. (2014). The gut microbiota, bacterial metabolites and colorectal cancer. Nature Reviews Microbiology, 12, 661–672. 10.1038/nrmicro3344 25198138

[fsn32421-bib-0096] Lunn, J., & Buttriss, J. L. (2010). Carbohydrates and dietary fibre. Nutrition Bulletin, 32, 21–64. 10.1111/j.1467-3010.2007.00616.x

[fsn32421-bib-0055] Macpherson, A. J., McCoy, K. D., Johansen, F. E., & Brandtzaeg, P. (2008). The immune geography of IgA induction and function. Mucosal Immunology, 1, 11–22. 10.1038/mi.2007.6 19079156

[fsn32421-bib-0056] Maier, T. V., Lucio, M., Lee, L. H., VerBerkmoes, N. C., Brislawn, C. J., Bernhardt, J., Lamendella, R., McDermott, J. E., Bergeron, N., Heinzmann, S. S., Morton, J. T., González, A., Ackermann, G., Knight, R., Riedel, K., Krauss, R. M., Schmitt‐Kopplin, P., & Jansson, J. K. (2017). Impact of dietary resistant starch on the human gut microbiome, metaproteome, and metabolome. Mbio, 8, e01343‐17. 10.1128/mBio.01343-17 29042495 PMC5646248

[fsn32421-bib-0057] Makki, K., Deehan, E. C., Walter, J., & Bäckhed, F. (2018). The impact of dietary fiber on gut microbiota in host health and disease. Cell Host & Microbe, 23, 705–715. 10.1016/j.chom.2018.05.012 29902436

[fsn32421-bib-0058] Martensson, O., Biorklund, M., Lambo, A. M., Duenas‐Chasco, M., Irastorz, A., Holst, O., Norin, E., Welling, G., Oste, R., & Onning, G. (2005). Fermented, ropy, oat‐based products reduce cholesterol levels and stimulate the Bifidobacteria flora in humans. Nutrition Research, 25, 429–442. 10.1016/j.nutres.2005.03.004

[fsn32421-bib-0059] Mentschel, J., & Claus, R. (2003). Increased butyrate formation in the pig colon by feeding raw potato starch leads to a reduction of colonocyte apoptosis and a shift to the stem cell compartment. Metabolism, 52, 1400–1405. 10.1016/s0026-0495(03)00318-4 14624397

[fsn32421-bib-0060] Montagne, L., Pluske, J. R., & Hampson, D. J. (2003). A review of interactions between dietary fibre and the intestinal mucosa, and their consequences on digestive health in young non‐ruminant animals. Animal Feed Science and Technology, 108, 95–117. 10.1016/S0377-8401(03)00163-9

[fsn32421-bib-0061] Montoya, C. A., Henare, S. J., Rutherfurd, S. M., & Moughan, P. J. (2016). Potential misinterpretation of the nutritional value of dietary fiber: Correcting fiber digestibility values for nondietary gut‐interfering material. Nutrition Reviews, 74, 517–533. 10.1093/nutrit/nuw014 27330145

[fsn32421-bib-0062] Montoya, C. A., Rutherfurd, S. M., & Moughan, P. J. (2017). Ileal digesta nondietary substrates from cannulated pigs are major contributors to in vitro human hindgut short‐chain fatty acid production. The Journal of Nutrition, 147, 264–271. 10.3945/jn.116.240564 28003537

[fsn32421-bib-0063] Morrison, D. J., & Preston, T. (2016). Formation of short chain fatty acids by the gut microbiota and their impact on human metabolism. Gut Microbes, 7, 189–200. 10.1080/19490976.2015.1134082 26963409 PMC4939913

[fsn32421-bib-0064] Mozaffarian, D., Hao, T., Rimm, E. B., Willett, W. C., & Hu, F. B. (2011). Changes in diets and lifestyle and long‐term weight gain in woman and men. The New England Journal of Medicine, 364, 2392–2404. 10.1056/NEJMoa1014296 21696306 PMC3151731

[fsn32421-bib-0065] Mu, C., Zhang, L., He, X., Smidt, H., & Zhu, W. (2017). Dietary fibres modulate the composition and activity of butyrate‐producing bacteria in the large intestine of suckling piglets. Antonie van Leeuwenhoek, 110, 687–696. 10.1007/s10482-017-0836-4 28161736

[fsn32421-bib-0066] Mudgil, D., & Barak, S. (2013). Composition, properties and health benefits of indigestible carbohydrate polymers as dietary fiber: A review. International Journal of Biological Macromolecules, 61, 1–6. 10.1016/j.ijbiomac.2013.06.044 23831534

[fsn32421-bib-0067] Natividad, J. M., & Verdu, E. F. (2013). Modulation of intestinal barrier by intestinal microbiota: Pathological and therapeutic implications. Pharmacological Research, 69, 42–51. 10.1016/j.phrs.2012.10.007 23089410

[fsn32421-bib-0068] Nicolucci, A. C., Hume, M. P., Martínez, I., Mayengbam, S., Walter, J., & Reimer, R. A. (2017). Prebiotics reduce body fat and alter intestinal microbiota in children who are overweight or with obesity. Gastroenterology, 153, 711–722. 10.1053/j.gastro.2017.05.055 28596023

[fsn32421-bib-0069] Nielsen, T. S., Lærke, H. N., Theil, P. K., Sørensen, J. F., Saarinen, M., Forssten, S., & Knudsen, K. E. (2014). Diets high in resistant starch and arabinoxylan modulate digestion processes and SCFA pool size in the large intestine and faecal microbial composition in pigs. British Journal of Nutrition, 112, 1837–1849. 10.1017/S000711451400302X 25327182

[fsn32421-bib-0070] Nielsen, T. S., Theil, P. K., Purup, S., Nørskov, N. P., & Bach Knudsen, K. E. (2015). Effects of resistant starch and arabinoxylan on parameters related to large intestinal and metabolic health in pigs fed fat‐rich diets. Journal of Agricultural and Food Chemistry, 63, 10418–10430. 10.1021/acs.jafc.5b03372 26566722

[fsn32421-bib-0071] Nilsson, A. C., Johansson‐Boll, E. V., & Björck, I. M. (2015). Increased gut hormones and insulin sensitivity index following a 3‐d intervention with a barley kernel‐based product: A randomised cross‐over study in healthy middle‐aged subjects. The British Journal of Nutrition, 114, 899–907. 10.1017/S0007114515002524 26259632

[fsn32421-bib-0072] Owusu‐Asiedu, A., Patience, J. F., Laarveld, B., Van Kessel, A. G., Simmins, P. H., & Zijlstra, R. T. (2006). Effects of guar gum and cellulose on digesta passage rate, ileal microbial populations, energy and protein digestibility, and performance of grower pigs. Journal of Animal Science, 84, 843–852. 10.2527/2006.844843x 16543561

[fsn32421-bib-0073] Pedersen, C., Lefevre, S., Peters, V., Patterson, M., Ghatei, M. A., Morgan, L. M., & Frost, G. S. (2013). Gut hormone release and appetite regulation in healthy non‐obese participants following oligofructose intake. A dose‐escalation study. Appetite, 66, 44–53. 10.1016/j.appet.2013.02.017 23474087

[fsn32421-bib-0074] Pieper, R., Jha, R., Rossnagel, B., Van Kessel, A. G., Souffrant, W. B., & Leterme, P. (2008). Effect of barley and oat cultivars with different carbohydrate compositions on the intestinal bacterial communities in weaned piglets. FEMS Microbiology Ecology, 66, 556–566. 10.1111/j.1574-6941.2008.00605.x 19049653

[fsn32421-bib-0075] Pryde, S. E., Duncan, S. H., Hold, G. L., Stewart, C. S., & Flint, H. J. (2002). The microbiology of butyrate formation in the human colon. FEMS Microbiology Letters, 217, 133–139. 10.1111/j.1574-6968.2002.tb11467.x 12480096

[fsn32421-bib-0076] Rea, M. C., Alemayehu, D., Ross, R. P., & Hill, C. (2013). Gut solutions to a gut problem: Bacteriocins, probiotics and bacteriophage for control of Clostridium difficile infection. Journal of Medical Microbiology, 62, 1369–1378. 10.1099/jmm.0.058933-0 23699066

[fsn32421-bib-0077] Rejaii, M., & Salehi, E. A. (2016). Properties of sugar beet pulp pectin: A systemic review. International Journal of PharmTech Research, 9, 364–368.

[fsn32421-bib-0095] Sajilata, M. G., Singhal, R. S., & Kulkarni, P. R. (2006). Resistant starch‐A review. Comprehensive Reviews in Food Science and Food Safety, 1, 1–17. 10.1111/j.1541-4337.2006.tb00076.x 33412740

[fsn32421-bib-0078] Sasaki, H., Miyakawa, H., Watanabe, A., Nakayama, Y., Lyu, Y., Hama, K., & Shibata, S. (2019). Mice microbiota composition changes by inulin feeding with a long fasting period under a two‐meals‐per‐day schedule. Nutrients, 11, 2802. 10.3390/nu11112802 PMC6893728 31744168

[fsn32421-bib-0079] Schiavon, S., Bortolozzo, A., Bailoni, L., & Tagliapetra, F. (2004). Effects of sugar beet pulp on growth and health status of weaned piglets. Italian Journal of Animal Science, 4, 337–351. 10.4081/ijas.2004.337

[fsn32421-bib-0080] Schulze, M. B., Liu, S., Rimm, E. B., Manson, J. E., Willett, W. C., & Hu, F. B. (2004). Glycemic index, glycemic load, and dietary fiber intake and incidence of type 2 diabetes in younger and middle‐aged women. American Journal of Clinical Nutrition, 80, 348–356. 10.1093/ajcn/80.2.348 15277155

[fsn32421-bib-0081] Serena, A., Hedemann, M. S., & Bach Knudsen, K. E. (2008). Influence of dietary fiber on luminal environment and morphology in the small and large intestine of sows. Journal of Animal Science, 86, 2217–2227. 10.2527/jas.2006-062 18310497

[fsn32421-bib-0082] Silva, F. M., Kramer, C. K., Crispim, D., & Azevedo, M. J. (2015). A high‐glycemic index, low‐fiber breakfast affects the postprandial plasma glucose, insulin, and ghrelin responses of patients with type 2 diabetes in a randomized clinical trial. Journal of Nutrition, 145, 736–741. 10.3945/jn.114.195339 25833777

[fsn32421-bib-0083] Smith, P. M., Howitt, M. R., Panikov, N., Michaud, M., Gallini, C. A., Bohlooly‐Y, M., Glickman, J. N., & Garrett, W. S. (2013). The microbial metabolites, short‐chain fatty acids, regulate colonic Treg cell homeostasis. Science, 341, 569–573. 10.1126/science.1241165 23828891 PMC3807819

[fsn32421-bib-0084] Stack, H. M., Kearney, N., Stanton, C., Fitzgerald, G. F., & Ross, R. P. (2010). Association of beta‐glucan endogenous production with increased stress tolerance of intestinal lactobacilli. Applied and Environmental Microbiology, 76, 500–507. 10.1128/AEM.01524-09 19933353 PMC2805207

[fsn32421-bib-0085] Tan, J., McKenzie, C., Potamitis, M., Thorburn, A. N., Mackay, C. R., & Macia, L. (2014). The role of short‐chain fatty acids in health and disease. Advances in Immunology, 121, 91–119. 10.1016/B978-0-12-800100-4.00003-9 24388214

[fsn32421-bib-0086] Thangaraju, M., Cresci, G. A., Liu, K., Ananth, S., Gnanaprakasam, J. P., Browning, D. D., Mellinger, J. D., Smith, S. B., Digby, G. J., Lambert, N. A., Prasad, P. D., & Ganapathy, V. (2009). GPR109A is a G‐protein‐coupled receptor for the bacterial fermentation product butyrate and functions as a tumor suppressor in colon. Cancer Research, 69, 2826–2832. 10.1158/0008-5472.CAN-08-4466 19276343 PMC3747834

[fsn32421-bib-0087] Tolhurst, G., Heffron, H., Lam, Y. S., Parker, H. E., Habib, A. M., Diakogiannaki, E., Cameron, J., Grosse, J., Reimann, F., & Gribble, F. M. (2012). Short‐chain fatty acids stimulate glucagon‐like peptide‐1 secretion via the G‐protein‐coupled receptor FFAR2. Diabetes, 61, 364–371. 10.2337/db11-1019 22190648 PMC3266401

[fsn32421-bib-0088] Venn, B. J., & Mann, J. (2004). Cereal grains, legumes and diabetes. European Journal of Clinical Nutrition, 58, 1143–1161. 10.1038/sj.ejcn.1601995 15162131

[fsn32421-bib-0089] Vila, F. M. (2017). Effects of dietary fiber on swine intestinal epithelial and immune response. Master's Thesis, University of Minnesota, Minnesota.

[fsn32421-bib-0090] Williams, B. A., Verstegen, M. W., & Tamminga, S. (2001). Fermentation in the large intestine of single‐stomached animals and its relationship to animal health. Nutrition Research Reviews, 14, 207–228. 10.1079/NRR200127 19087424

[fsn32421-bib-0091] Zhao, J., Bai, Y., Tao, S., Zhang, G., Wang, J., Liu, L., & Zhang, S. (2019). Fiber‐rich foods affected gut bacterial community and short‐chain fatty acids production in pig model. Journal of Functional Foods, 57, 266–274. 10.1016/j.jff.2019.04.009

[fsn32421-bib-0092] Zhao, J., Bai, Y., Zhang, G., Liu, L., & Lai, C. (2020). Relationship between dietary fiber fermentation and volatile fatty acids' concentration in growing pigs. Animals, 10, 263. 10.3390/ani10020263 PMC7070776 32045993

[fsn32421-bib-0093] Zhao, J., Liu, P., Wu, Y., Guo, P., Liu, L., Ma, X., Levesque, C., Chen, Y., Zhao, J., Zhang, J., & Ma, X. (2018). Dietary fiber increases butyrate‐producing bacteria and improves the growth performance of weaned piglets. Journal of Agricultural and Food Chemistry, 66, 7995–8004. 10.1021/acs.jafc.8b02545 29986139

[fsn32421-bib-0094] Zhou, L. P., Fang, L. D., Sun, Y., & Zhu, W. (2016). Effects of a diet high in resistant starch on fermentation end‐products of protein and mucin secretion in the colons of pigs. Starch, 69, 7–8. 10.1056/nejmoa1014296

